# Occurrence of Hybrid Diarrhoeagenic *Escherichia coli* Associated with Multidrug Resistance in Environmental Water, Johannesburg, South Africa

**DOI:** 10.3390/microorganisms9102163

**Published:** 2021-10-17

**Authors:** John Y. Bolukaoto, Atheesha Singh, Ntando Alfinete, Tobias G. Barnard

**Affiliations:** Water and Health Research Centre, University of Johannesburg, Doornfontein 2092, South Africa; jbolukaoto@uj.ac.za (J.Y.B.); asingh@uj.ac.za (A.S.); nalfinete@uj.ac.za (N.A.)

**Keywords:** diarrhoeagenic *Escherichia coli*, virulence gene, hybrid pathotypes, environmental water, South Africa

## Abstract

This study was undertaken to determine the virulence and antibiotic resistance profiles of diarrhoeagenic *Escherichia coli* (DEC) in environmental waters of Johannesburg, South Africa. Samples were collected and cultured on selective media. An 11-plex PCR assay was used to differentiate five DEC, namely: enteroaggregative (EAEC), enterohaemorrhagic (EHEC), enteroinvasive (EIEC), enteropathogenic (EPEC) and enterotoxigenic (ETEC). The antibiotic resistance profile of isolates was determined using the VITEK^®^-2 automated system. The virulence profiles of 170 *E. coli* tested showed that 40% (68/170) were commensals and 60% (102/170) were pathogenic. EPEC had a prevalence of 19.2% (32/170), followed by ETEC 11.4% (19/170), EAEC 6% (10/170) and EHEC 3% (5/170). Hybrid DEC carrying a combination of simultaneously two and three pathogenic types was detected in twenty-eight and nine isolates, respectively. The antibiotic susceptibility testing showed isolates with multidrug resistance, including cefuroxime (100%), ceftazidime (86%), cefotaxime (81%) and cefepime (79%). This study highlighted the widespread occurrence of DEC and antibiotic resistance strains in the aquatic ecosystem of Johannesburg. The presence of hybrid pathotypes detected in this study is alarming and might lead to more severe diseases. There is a necessity to enhance surveillance in reducing the propagation of pathogenic and antibiotic-resistant strains in this area.

## 1. Introduction

Diarrhoea is one of the common causes of morbidity and mortality among infants and children in most developing countries [1]. It is estimated that diarrhoea causes 526,000 deaths of children younger than five per year [2,3]. *Escherichia coli* (*E. coli*) is an anaerobic Gram-negative, typically rod-shaped bacterium considered as part of the normal flora of the gut of humans and warm-blooded animals, and can also be found in the environment, e.g., in water and soil [4,5]. According to its biological significance, *E. coli* is classified into harmless commensals, pathogenic and extra-intestinal pathogenic strains [6]. Pathogenic or diarrhoeagenic *Escherichia coli* (DEC) strains are among the causative agents of diarrhoea outbreaks and other serious waterborne and foodborne infections in humans [7,8]. Seven major DEC have been described: (i) adherent-invasive *E. coli* (AIEC), (ii) diffusely adherent *E. coli* (DAEC), (iii) enteroaggregative *E. coli* (EAEC), (iv) enterohaemorrhagic *E. coli* (EHEC), (v) enteroinvasive *E. coli* (EIEC), (vi) enteropathogenic *E. coli* (EPEC) and (vii) enterotoxigenic *E. coli* (ETEC) [9,10,11]. These pathotypes are classified based on clinical features, epidemiological evidence, phenotypic traits and specific virulence factors [12,13].

EAEC is a pathotype associated with persistent diarrhoea in humans and has been identified as possessing a plasmid of aggregative adhesion (*pAA*), for fimbriae production, which contains the aggregative adherence fimbriae type R (*agg*R) [12]. EHEC is an important enteric pathogen that produces Shiga toxin and causes a variety of clinical syndromes in humans, including bloody and non-bloody diarrhoea, haemorrhagic colitis and haemolytic uremic syndrome (HUS) [14,15]. EIEC is a pathotype that possesses invasion plasmid antigen H (*ipa*H) and invasive antigen locus (*ial*) genes, which encode the virulence regulon, the key for severe/invasive intestinal infections and dysentery [16,17]. EPEC and ETEC cause significant diarrhoeal illness and mortality in children, mostly in the developing world. EPEC strains are classified as typical or atypical, according to the presence or absence of the *E. coli* adherence factor plasmid (EAF) that carries the *bfpA* gene, which encodes for the bundle-forming pili [18]. Typical EPEC (tEPEC) contain both the locus of enterocyte effacement (LEE) region for attaching and effacing lesion (*eaeA* gene) and the *bfpA* gene, while atypical EPEC (aEPEC) are strains that do not contain the *bfpA* gene [19]. ETEC is a pathotype that colonises the small intestine and causes watery diarrhoea, known as traveller’s diarrhoea, in humans by producing plasmid-encoded heat-stable (ST) and/or heat-labile enterotoxin (LT) [20].

Hybrid DEC harbouring a combination of virulence genes have emerged worldwide and have been reported as a public health concern [21,22]. This is due to horizontal gene transfer (HGT) among diarrhoeagenic groups of *E. coli* [22,23] or to the fact that most *E. coli* virulence genes are generally found on plasmids, transmissible by means of conjugation [24]. The first hybrid pathotype (EAEC/EHEC or EAHEC) with genetic recombination was reported during a German diarrhoea outbreak [25]. Since then, several other studies have reported molecular evidence and genomics of hybrid pathotypes worldwide, including EPEC/ETEC in India [21] and in the USA [19], EPEC/EHEC in Brazil [14] and STEC/ETEC in Sweden [13,22].

The widespread occurrence of waterborne infections and the increase in antibiotic-resistant strains have become global health concerns. According to the Centres for Disease Control and Prevention, at least 2.8 million people acquire an antibiotic-resistant infection, and more than 35,000 people die each year in the United States alone [26]. In addition to its role as an indicator of microbiological purity of water, *E. coli* is also widely recognised due to its role in spreading antibiotic resistance in the water environment [27]. Several studies have reported antimicrobial-resistant strains’ transmission between animals, humans and water environments, including streams, rivers and lakes as well as from discharge that flows from hospitals, farms or sewage systems [28,29,30]. Understanding the different ways that antimicrobial resistance-determining genes spread and their transmission between various components of the ecosystem will contribute to the development of new concepts to prevent this process [31]. Studies have described several antibiotic resistance mechanisms in *E. coli* bacteria, including conjugation and horizontal gene transfer among the isolates [5,27]. However, the level of risks caused by antimicrobial-resistant strains to human health is still not fully documented.

In South Africa, only a few studies have reported the virulence and antibiotic resistance profile of DEC isolated in the environment. Therefore, this study aimed to determine the virulence profile of five DEC with special attention to the hybrid strains with the potential genetic combinations and their antibiotic resistance profile in the environmental water of Johannesburg, South Africa.

## 2. Materials and Methods

### 2.1. Ethical Consideration and Sample Collection

**Figure 1:**
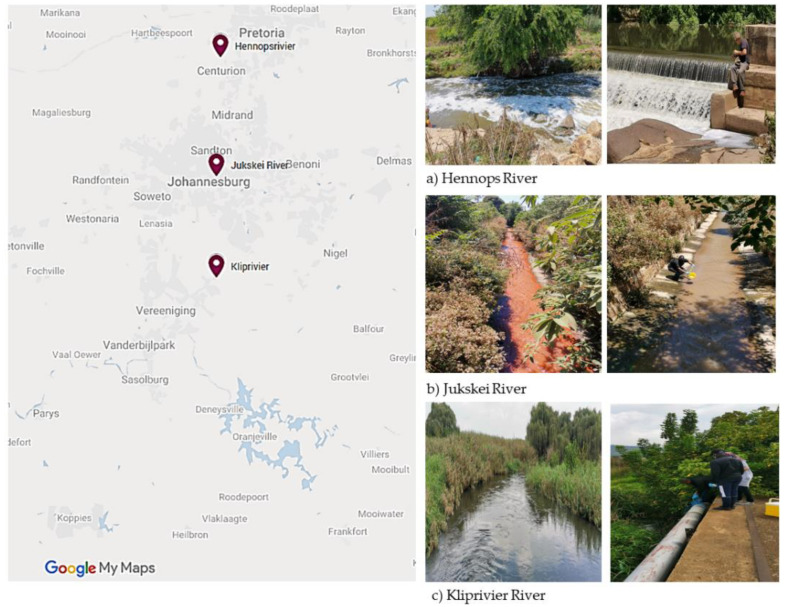
Map of the water sampling sites for study areas within the Johannesburg region. Shown are the locations’ approximate sampling points of the (**a**) Hennops, (**b**) Jukskei and (**c**) Kliprivier Rivers investigated in this study.

Ethical clearance to conduct this study was obtained from the Research Ethics Committees, Faculty of Health Sciences, University of Johannesburg (REC-168-2019), before sample collection. Environmental water samples (*n* = 101) were collected from nine water sources between August 2020 and February 2021. These included: (i) surface water from Jukskei River (*n* = 46) and Kliprivier River (Eikenhof) (*n* = 33), (ii) run-off water, upstream and downstream from the six Hennops river sites (*n* = 19), and (iii) sewage water from a stream and a well (*n* = 3), all in the Johannesburg, Gauteng Province, South Africa (as shown in Figure 1). Water samples were collected aseptically in sterile 1 litre glass bottles and properly labelled, and the temperature (°C) and potential of hydrogen ion (pH) were tested in situ by using a waterproof tester (Hanna, Sigma-Aldrich, Saint Louis, MI, USA). Samples were transported in an icebox to the laboratory of Water and Health Research Centre, the University of Johannesburg, for processing within 4 h.

### 2.2. Bacterial Isolation 

*Escherichia coli* were recovered from the water samples by a standard membrane filtration procedure. Briefly, 100 mL of water samples were serially diluted and filtered through 0.45 μm S-PAK^®^ membrane filters (Millipore, Sigma-Aldrich, Darmstadt, Germany). The membrane filters were aseptically placed directly onto HiChrome^®^ Coliform chromogenic media (Sigma-Aldrich, St. Louis, MO, USA) and incubated (Scientific Series 2000, USA) at 37 °C for 18 to 24 h. Bluish colonies were selected as presumptive *E. coli* (as indicated on the manufacturer’s manual) and were sub-cultured onto Müller-Hinton agar plates (Oxoid, UK) and re-incubated at 37 °C for 24 h. Pure colonies were Gram-stained and identified using the VITEK-2^®^ automated system (bioMérieux, Marcy-l’Étoile, France). *Escherichia coli* isolates were inoculated into Luria Bertani (LB) broth (HiMedia^®^ Laboratories Pvt. Ltd., India), grown overnight (at 37 °C for 24 h) and stored long-term at −80 °C in a biofreezer (ThermoScientific, Waltham, MA, USA) in a 50% (*v*/*v*) sterile glycerol solution (Associated Chemical Enterprises (Pty) Ltd., Gauteng, South Africa) until further analysis. 

### 2.3. DNA Extraction from Escherichia coli Isolates

Two mL of presumptive isolates were grown in LB broth (HiMedia^®^ Laboratories Pvt. Ltd., Maharashtra, India) at 37 °C overnight and the total genomic DNA was extracted using the silica/guanidium thiocyanate (Sigma-Aldrich, Saint Louis, MI, USA) method adapted from the article previously published [32]. The extracted DNA was quantified using the Nanodrop instrument (Jenway Genova Nano, USA).

### 2.4. Multiplex PCR for the Detection of Virulence Profile of Escherichia coli

A single-step 11-gene multiplex PCR assay was performed on *E. coli* isolates for the detection of the virulence genes based on the methods and conditions previously described [28], using primer sequences targeting the *E. coli* genes presented in Table 1. All the primers used in this study were synthesised by WhiteHead Scientific (Pty) Ltd., South Africa.

The 20 μL Hotstart multiplex PCR reaction consisted of 10 μL of Qiagen master mix (Qiagen, Germany), 1 μL of the primer mixture (forward and reverse) (0.1 μM of *lt* primers, 0.2 μM of *asta*, *bfp, eagg*, *ial*, and *gapdh* primers, 0.3 μM of *eaeA* and *stx2* primers, 0.5 μM of *stx1* and *sta* primers)), 2 μL of M_g_Cl_2_, 1 μL of Q-solution, 4.0 μL of PCR-grade water and 2 μL of sample DNA. The following conditions were used during PCR amplification performed in a Bio-Rad MyCycler^TM^ Thermal cycler (Bio-Rad, Hercules, CA, USA): an initial activation at 95 °C for 15 min, followed by 35 cycles consisting of denaturation at 94 °C for 45 s, annealing at 55 °C for 45 s, extension at 68 °C for 2 min and final extension at 72 °C for 5 min.

### 2.5. Singleplex PCR for the Confirmation of Escherichia coli 

A singleplex PCR assay was performed for the confirmation of genes present in each positive *E. coli* isolate using the primer sequences presented in Table 1. The 20 μL PCR reaction consisted of 2 μL of 10× buffer (Qiagen, Germany), 2 μL of the primer mixture (forward and reverse), 2 μL of MgCl_2_, 4 μL of Q-solution, 0.4 μL of dNTPs, 0.1 μL of Taq polymerase, 7.5 μL of PCR-grade water and 2 μL of genomic DNA. The PCR conditions were the same as described in Section 2.4.

### 2.6. Visualisation of PCR Products

The PCR-amplified products were separated on a 2.5% (*m*/*v*) agarose gel (Bioline, Taunton, MA, USA), and stained with 5 µL of a 10 mg/mL stock solution of ethidium bromide (Merck, New York, NY, USA) using TAE buffer (40 mM Tris-acetate, 2 mM EDTA, pH 8.3). Gel electrophoresis was performed at 90 volts for 120 min. A 100 bp molecular weight marker (Fermentas, Thermo Scientific, Waltham, MA, USA) was included as a reference in all gels. The gel images were visualised and captured using a Gel Doc^TM^ EZ system (Bio-Rad, Hercules, CA, USA).

### 2.7. Control Strains and Data Analysis

The control strains used in this study included *E. coli:* (Commensal) ATCC 25922, enteroinvasive (EIEC) ESCCOS ATCC 43893, enteroaggregative (EAEC) ESCCO 14, enterohaemorrhagic (EHEC) ESCCO 21, enterotoxigenic (ETEC) ESCCO 22 and enteropathogenic (EPEC) S-ESCCO 16 Pl. All these strains were purchased from the National Health Laboratory Service (NHLS), South Africa, and were confirmed in a previous study [28]. The positive m-PCR was made up (in-house) by combining the extracted plasmids that contained the targeted virulence genes (*eaeA, eagg, Asta, bfp, gapdh, ial, lt, mdh, sta, stx1, stx2*). The *malate dehydrogenase* (*mdh*) housekeeping gene was used as an internal control and the *glyceraldehyde 3-phosphate dehydrogenase* (*gapdh*) gene was used as an external control. The gel images were analysed by reading the presence of bands detected. All data obtained were recorded and exported into a Microsoft Excel sheet for analysis.

### 2.8. Antibiotic Susceptibility Testing of Escherichia coli Isolates

All pathogenic *Escherichia coli* and some commensal isolates were selected and tested for antibiotic susceptibility using the VITEK^®^2 automated system (bioMérieux, Marcy-l’Étoile, France). Briefly, a bacterial suspension with an optical density (turbidity) of 0.5 McFarland was prepared in saline (0.85%) (bioMérieux, France) from an overnight bacterial culture incubated (Vacutec, Roodepoort, South Africa) for 18 h. The antibiotics included in the VITEK^®^2 automated system panel were: ampicillin, amikacin, amoxicillin/clavulanic acid, cefepime, cefotaxime, cefoxitin, ceftazidime, cefuroxime, cefuroxime-Axetil, ciprofloxacin, colistin, ertapenem, gentamicin, imipenem, meropenem, piperacillin/tazobactam, tigecycline, tobramycin and trimethoprim/sulfamethoxazole. The minimum inhibitory concentration (MIC) for each isolate tested was interpreted according to the Clinical and Laboratory Standards Institute (CLSI) 2021 guidelines [35].

## 3. Results

### 3.1. Isolation and Detection of E. coli Isolates from Environmental Water Samples 

**Figure 2:**
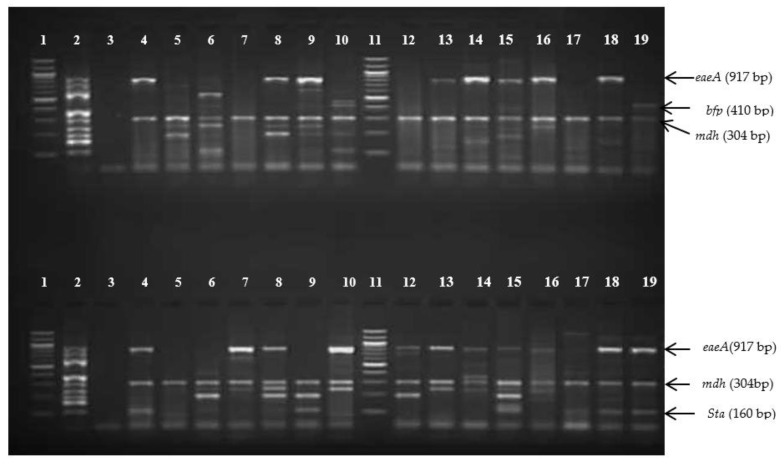
Gel image of m-PCR products of *E. coli* isolates: **Top** and **Bottom** wells: Lanes 1 and 11: 100 bp molecular marker (ladder), Lane 2: positive control, Lane 3: negative control, Lanes 4–10 and Lanes 12–19: *E. coli* isolates showing different genes.

In total, 101 environmental water samples were collected and analysed in this study. The HiChrome^®^ Coliform chromogenic media (Sigma-Aldrich, Saint Louis, MI, USA) identified 288 presumptive *E. coli*, which appeared bluish, as from the manufacturer. The PCR assay confirmed 170 *E. coli* isolates with the detection of the *mdh* housekeeping gene. The presence of the *gapdh* gene used as an external control excluded any possible PCR inhibition in all samples. The 11-gene multiplex PCR assays indicated that 40% (68/170) were commensals *E. coli* (ComEC) and 60% (102/170) were positive for at least one pathogenic *E. coli* type. Figure 2 shows the gel image of m-PCR with different genes detected in this study. 

### 3.2. Detection of Pathogenic Types of E. coli Isolates by Multiplex PCR

#### 3.2.1. *Escherichia coli* (Single Pathogenic Type) Detected in This Study

Enteropathogenic *E. coli* (EPEC) was the most detected in this study, with 19.2% (32/170) of isolates, of which atypical EPEC (aEPEC) harbouring only the *eaeA* gene accounted for 18% (30/170) while typical EPEC (tEPEC) harbouring both the *eaeA* and *bfp* genes accounted for 0.6% (1/170), and the *bfp* gene alone was present in 0.6% (1/170). Enterotoxigenic *E. coli* was detected in 11.4% (19/170) of isolates, with only a limited number of isolates harbouring both *lt* and *sta* genes (1.2%, 2/170), while *sta* alone was present in 9% (15/170) and *lt* alone in 1.2% (2/170). EAEC harbouring the *eagg* gene was present in 6% (10/170), and EHEC was detected in 3% (5/170), of which 1.2% (2/170) of isolates harboured *stx*1 and *stx*2 each, whereas both *stx*1 and *stx*2 were detected in 0.6% (1/170). EIEC harbouring the *ial* gene was not detected in this study. Table 2 shows the details of single *E. coli* pathotypes and their virulence genes detected.

#### 3.2.2. Hybrid Pathogenic Types (Two Pathotypes) Detected in This Study

Hybrid pathogenic types forming two pathotypes harbouring a combination of virulent genes were detected in 28 isolates, including EAEC/EPEC (7.6%, 13/170), EAEC/ETEC (3%, 5/170), EPEC/ETEC (2.4%, 4/170), EPEC/EHEC (1.8%, 3/170) and EHEC/ETEC (1.8%, 3/170). The complete distribution of virulence genes forming a hybrid of two pathotypes is illustrated in Table 3.

#### 3.2.3. Hybrid Pathogenic Types (Three Pathotypes) Detected in This Study

A hybrid of three different pathotypes simultaneously harbouring a combination of virulent genes was detected in nine isolates, including EAEC/aEPEC/ETEC (2.4%, 4/170), EHEC/ETEC/aEPEC (1.8%, 3/170) and EAEC/EHEC/ETEC (1.2%, 2/170). Table 4 shows the full distribution of virulence genes identified in *E. coli* isolates forming three pathotypes. Singleplex PCRs were run on all hybrid isolates as confirmatory tests and the results showed the presence of the single genes detected by different pathogenic types (Supplementary Materials).

#### 3.2.4. Antimicrobial Susceptibility Testing Results of *Escherichia coli* Isolates 

**Figure 3:**
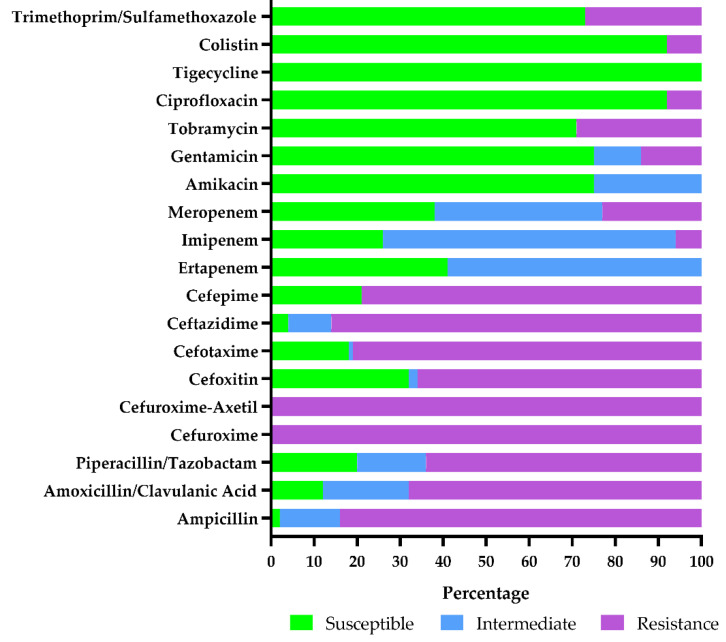
Antibiotic resistance patterns of *E. coli* isolates (*n* = 100) using the VITEK^®^2 automated system (bioMérieux, Marcy-l’Étoile, France) in Johannesburg, South Africa.

Antibiotic resistance patterns of selected *E. coli* isolates (*n* = 100) using the VITEK^®^2 automated system (bioMérieux, France) showed isolates with multidrug resistance (Figure 2). All isolates were 100% (100/100) resistant to cefuroxime, 98% (98/100) to ampicillin and 96% (96/100) to ceftazidime. In total, 88%, 82%, 80%, 79% and 68% of isolates were resistant to amoxicillin/clavulanic acid, cefotaxime, piperacillin/tazobactam, cefepime and cefoxitin, respectively. Imipenem, meropenem and ertapenem resistance were 74%, 62% and 59%, respectively. Resistance to amikacin and gentamicin was observed in 25% (15/50) each. No tigecycline resistance was reported in this study. All intermediately resistant isolates were reported as resistant (Figure 3). At least 8% (8/100) of *E. coli* isolates showed an increased colistin MIC using the VITEK^®^2 automated system (bioMérieux, France). 

## 4. Discussion

Diarrhoea outbreaks are a persistent problem with significant economic and potential public health impacts worldwide. In most developing countries, people continue to use environmental water (river water and stream) for domestic activities such as bathing, washing clothes, cooking and drinking [36]. It has also been reported that the spread of bacteria in the environment can be affected by the discharge of municipal sewage into surface water and soil [27]. Consequently, waterborne pathogens including diarrhoeagenic *E. coli* (DEC) can pass from the environment to humans, causing severe diseases. Studies have reported waterborne diarrhoeal disease claiming two million deaths worldwide each year, mostly in children below 5 years of age [37].

This study was carried out to determine the virulence profile of DEC and their antibiotic resistance profile in the environmental water of the Johannesburg region, South Africa. In total, 101 samples were included in this study, from which 288 presumptive *E. coli* were identified using HiChrome^®^ Coliform selective chromogenic media (Sigma-Aldrich, USA) and the VITEK^®^-2 automated system (bioMérieux, France). Studies have reported these techniques to generate many errors and they can accommodate the growth of other species, leading to the misidentification of colonies, especially in environmental samples [38]. As such, molecular testing was performed on presumptive isolates as a confirmatory test. The single-step 11-gene multiplex PCR was used and identified DEC strains in environmental water. Overall, 170 *E. coli* were isolated, and the prevalence of commensal and DEC identified was 40% (68/170) and 60% (102/170), respectively. Among the DEC identified, EPEC was the most common single enteropathogen, with 19.2% (32/170) of cases. Studies have shown that infections due to EPEC are usually endemic in developing countries [37,39]. The proportion of EPEC detected in this study is consistent with other studies in South Africa [36,40] and elsewhere in the world [8,37,41]. In total, 18% (30/170) of isolates were atypical EPEC (aEPEC) harbouring the single gene *eaeA,* and 1.2% (2/170) were typical EPEC (tEPEC), of which 0.6% harboured both *eaeA* and *bfp* genes and 0.6% harboured the single *bfp* gene. The importance of distinguishing typical and atypical EPEC is that tEPEC causes infections mostly in infants, while aEPEC has been reported to cause infections in both children and adults [18]. The proportion of aEPEC in this study was lower compared to the 86% reported by Traoré et al. [42] in Vhembe district, Limpopo, South Africa. This difference might be due to the difference in geographic region and to the increase in human activity observed in environmental water of Vhembe district by villagers compared to the city of Johannesburg [42].

This study detected ETEC in 11.4% (19/170) of isolates. The genes that encode both heat-liable (LT) and heat-stable (ST) enterotoxins in ETEC are generally found on plasmids, transmissible and causing severe diarrhoea [43]. Our finding on ETEC was lower than the 47% and 83% reported by Nontongana et al. [44] and Traoré et al. [42] in the rural areas of the Eastern Cape and Limpopo provinces of South Africa, respectively. However, the proportion of ETEC detected in this study correlated with the previous findings in the surface water of northwest Mexico [45].

This study has identified 6% (10/170) of EAEC pathotypes in environmental water. Infection due to the EAEC pathotype is dangerous in immuno-compromised individuals and children [36] and has also been reported as one of the leading causes of DEC-associated food- and water-borne enteric infection [46]. The prevalence of EAEC identified in this study correlated with the study by Tanih et al. [47]. However, this proportion was lower when compared to the 87%, 34.4% and 58.3% reported by Traoré et al. [36], Canizalez-Roman et al. [45] and Mbanga et al. [48], respectively.

The current study detected the EHEC pathotype in 3% (5/170). Two isolates harboured each single *stx*1 and *stx*2 gene, while one isolate harboured both *stx*1 and *stx*2. The detection of the *stx* gene in river water is of concern because the colonisation of the human large intestine with EHEC *stx* even in low proportions can result in potentially fatal complications, such as haemolytic uremic syndrome (HUS) [20]. Previous studies conducted in South Africa have reported EHEC rates of 15.0% [40], 15.08% [49] and 8.3% [47].

A similar study conducted in the USA has reported 14% of EHEC in water samples [38]. In Georgia, Cho and co-workers reported a low rate (0.2%) of EHEC in watershed [50]. In this study, EIEC harbouring *ial* virulence genes was not detected in environmental water. This is not surprising because this pathotype is mostly reported as causing dysentery in humans and sometimes in animals [16,51].

Our study revealed hybrid pathotypes with virulence combinations among the isolates. This might be due to the mechanism of conjugation in virulence-associated genes which define a pathotype and are carried on mobile genetic elements (plasmids) [20]. Hybrids of Shiga toxin-producing and enterotoxigenic *E. coli* (EHEC/ETEC) have previously been reported associated with diarrhoeal disease in humans and animals [24,52]. In the present study, hybrid EHEC/ETEC was detected in 1.8% of isolates. This is serious and should be considered for epidemiological surveillance. Similarly, studies have reported 2.05% and 34% of hybrid EHEC/ETEC in Sweden [13] and in Bangladesh [43], respectively. This current study reported hybrids EAEC/aEPEC in 7.6% (13/170). A similar situation has been reported in Mexico [53]. The present study detected hybrids EPEC/ETEC and EPEC/EHEC in 2.4% and 1.8%, respectively. In India, Dutta and co-workers [21] identified hybrid EPEC/ETEC in a child with acute diarrhoea, while EPEC/EHEC strains were detected in Iran [54] and Mexico [14]. All these strains detected are virulent and might contribute to severe diarrhoea outbreaks and could pose a potential public health threat to consumers of untreated environmental water. Studies have reported molecular evidence of such hybrid pathotypes in humans, animals and environmental origins.

Interestingly, in this study, three different DEC strains were simultaneously detected in isolates. Hybrids of aEPEC + EHEC + ETEC harbouring *eaeA + stx*1 *+ stx*2 *+ lt + sta* were detected in two isolates. This indicates that hypervirulent strains are circulating in our area. Further confirmatory tests such as sequencing and comparative genomics would need to be performed on these strains to characterise and determine their phylogenetic position. Similarly, in Finland, three hybrid pathotypes were detected among *E. coli* strains harbouring up to six gene products [55]; while in Mexico, Patzi-Vargas et al. [53] detected three different DEC strains (EAEC + ETEC + DAEC) in one isolate.

In recent times and in modern medicine, the increasing microbial drug resistance has been identified as the biggest public health challenge and listed as one of the public health priorities among CDC fights [26,27,56]. According to the Global Antimicrobial Resistance Surveillance System (GLASS) report, *E. coli* is one of the pathogens that cause common hospital-acquired and community-acquired infections worldwide. Consequently, treatment is becoming increasingly difficult due to high rates of antimicrobial resistance [57]. In this study, the antibiotic resistance profile of *E. coli* isolates was determined and resistance to multiple antibiotics was identified in the environmental water using the VITEK^®^2 automated system (bioMérieux, France). Resistance to the most common antibiotics used for the treatment of infections due to DEC, including last-resort antibiotics, was observed among the isolates. The most abundant resistances were against cefuroxime (100%) and both ampicillin and ceftazidime (94%), followed by cefotaxime (88%), cefepime (84%), amoxicillin/clavulanic acid (82%) and imipenem (70%). These findings are higher as compared to previous study findings in the KwaZulu-Natal (Durban), Western Cape (Stellenbosch) and Eastern Cape provinces of South Africa [28,30,48]. This is alarming and shows that antibiotic resistance strains are circulating in South Africa. A similar situation was observed in Poland, where numerous multidrug-resistant strains were detected in water samples, which did not exhibit the ESBL phenotype [27]. The presence of antibiotic-resistant bacteria in source water is considered to be an emerging health concern in humans [52]. To prevent the spread of drug resistance in the region, several actions should be taken, including the monitoring of antimicrobial consumption, measuring antibiotic use and antimicrobial resistance genes’ surveillance in the environment [57,58].

One of the limitations of this study was the use of the VITEK^®^-2 automated system to assess drug susceptibility. This phenotypic method is mostly used in routine diagnosis laboratories to determine the bacterial susceptibility/resistance to antimicrobials (which sometimes generates errors). It has also been reported that some of the VITEK^®^-2 cards’ susceptible MIC breakpoints differ from one another as recommended for therapy of some infections per CLSI [59]. Furthermore, the VITEK^®^-2 automated system does not detect the presence of antibiotic-resistant genes as performed in scientific research laboratories. Further tests including sequencing would need to be performed on these isolates for the detection of extended-spectrum beta-lactamases, carbapenemases and plasmid-mediated colistin antibiotic resistance genes.

## 5. Conclusions

This present study highlighted the widespread occurrence of potentially DEC and antibiotic resistance genes in the aquatic ecosystem of Johannesburg, South Africa. Among the enteropathogens tested, EPEC was the most dominant, followed by ETEC, EAEC and EHEC. The presence of hybrid pathotypes detected in this study can pose a potential public health risk to consumers of untreated water in the region. The single-step 11-gene multiplex PCR system used in this study is potentially a quick, powerful and useful method for routine monitoring, virulence gene screening and risk assessment of water quality in developing countries. This study reported the presence of numerous multidrug-resistant strains among the isolates, of which resistance to the most common antibiotics used for the treatment of infections due to DEC was observed. There is a necessity to enhance surveillance in reducing the propagation of pathogenic and antibiotic-resistant *E. coli* bacteria, which are environmental and public health concerns. 

## Figures and Tables

**Table 1:** Primers used for multiplex PCR in this study.

Pathogen	Gene Primers	Primer Sequence (5′– 3′)	Size (bp)	Reference
Internal control	*mdh (F)*	GGT ATG GAT CGT TCC GAC CT	304	[32]
	*mdh (R)*	GGC AGA ATG GTA ACA CCA GAG T		
External control	*gapdh (F)*	GAG TCA ACG GAT TTG GTC GT	238	[33]
	*gapdh (R)*	TTG ATT TTG GAG GGA TCT CG		
EIEC	*ial (F)*	GGT ATG ATG ATG ATG AGT CCA	650	[34]
	*ial (R)*	GGA GGC CAA CAA TTA TTT CC		
EHEC	*stx1 (F)*	ACA CTG GAT GAT CTC AGT GG	614	[32]
	*stx1 (R)*	CTG AAT CCC CCT CCA TTA TG		
	*stx2 (F)*	CCA TGA CAA CGG ACA GCA GTT	779	
	*stx2 (R)*	CCT GTC AAC TGA GCA CTT TG		
EHEC/aEPEC	*eaeA (F)*	CTG AAC GGC GAT TAC GCG AA	917	[34]
	*eaeA (R)*	CCA GAC GAT ACG ATC CAG		
tEPEC	*bfpA (F)*	AAT GGT GCT TGC GCT TGC TGC	410	
	*bfpM (R)*	TAT TAA CAC CGT AGC CTT TCG CTG AAG TAC CT		
EAEC	*eagg (F)*	AGA CTC TGG CGA AAG ACT GTA TC	194	[34]
	*eagg (R)*	ATG GCT GTC TGT AAT AGA TGA GAA C		
ETEC	*lt-1 (F)*	TGG ATT CAT CAT GCA CCA CAA GG	360	[34]
	*lt-1 (R)*	CCA TTT CTC TTT TGC CTG CCA TC		
	*sta (F)*	TTT CCC CTC TTT TAG TCA GTC AAC TG	160	
	*sta(R)*	GGC AGG ATT ACA ACA AAG TTC ACA		
*E. coli* toxin	*astA (F)*	GCC ATC AAC ACA GTA TAT CC	106	[32]
	*astA (R)*	GAG TGA CGG CTT TGT AGT C		

**Table 2:** *Escherichia coli* (single pathotype) and their virulence genes detected in this study.

Pathogenic Types	Genes Detected	*n* (170)	%
**ComEC**	*mdh*	68	40
**EPEC**		32	19.2
**aEPEC**	*eaeA*	(30)	(18.0)
**tEPEC**	*bfp*	(1)	(0.6)
	*bfp + eaeA*	(1)	(0.6)
**ETEC**		19	11.4
	*lt*	(2)	(1.2)
	*sta*	(15)	(9.0)
	*lt + sta*	(2)	(1.2)
**EHEC**		5	3.0
	*stx1*	(2)	(1.2)
	*stx2*	(2)	(1.2)
	*stx1 + stx2*	(1)	(0.6)
**EAEC**	*eagg*	10	6.0
**EIEC**	*ial*	0	0.0

*n*: number of isolates; ComEC: commensal *E. coli*; aEPEC: atypical EPEC; tEPEC: typical EPEC.

**Table 3:** *Escherichia coli* hybrid pathogenic types (two pathotypes) and their virulence genes detected in this study.

Hybrid Pathotypes	Gene Combinations Detected	*n* (170)	%
**EAEC/aEPEC**	*eaeA + eagg*	13	7.6
**EAEC/ETEC**		5	3.0
	*eagg + sta*	(3)	(1.8)
	*eagg + lt + sta*	(2)	(1.2)
**EPEC/ETEC**		4	2.4
	*eaeA + lt*	(1)	(0.6)
	*eaeA + lt + sta*	(3)	(1.8)
**EHEC/ETEC**		3	1.8
	*Stx*1 + *sta*	(1)	(0.6)
	*Stx*2 + *sta*	(1)	(0.6)
	*Stx*1 + *lt* + *sta*	(1)	(0.6)
**EPEC/EHEC**		3	1.8
**aEPEC/EHEC**	*eaeA + stx2*	(2)	(1.2)
**tEPEC/EHEC**	*eaeA + bfp + stx*1 *+ stx*2	(1)	(0.6)

*n*: number of isolates.

**Table 4:** Hybrid pathogenic type (three pathotypes) of *E. coli* and their virulence genes detected in this study.

Hybrid Pathotypes	Gene Combinations Detected	*n* (170)	%
**EAEC/aEPEC/ETEC**	*eagg + eaeA + sta*	4	2.4
**EAEC/EHEC/ETEC**	*eagg + stx*1 *+ sta*	2	1.2
**EHEC/ETEC/aEPEC**		3	1.8
	*eaeA + stx*1 *+ sta*	(1)	(0.6)
	*eaeA + stx1 + stx*2 *+lt + sta*	(2)	(1.2)

## Data Availability

All data generated or analyzed during this study are included in this article and its Supplementary Materials files.

## Supplementary Materials

The following are available online at https://www.mdpi.com/article/10.3390/microorganisms9102163/s1.
