# Supplementary material for: Occurrence of Hybrid Diarrhoeagenic Escherichia coli Associated with Multidrug Resistance in Environmental Water, Johannesburg, South Africa
Source: Microorganisms. 2021 Oct 17;9(10):2163. doi: 10.3390/microorganisms9102163 (PMC8538365; doi:10.3390/microorganisms9102163)
Supplement: Supplementary file 1 [file microorganisms-09-02163-s001.zip › microorganisms-1377333-supplementary.pdf]

S1. Gel images of singleplex PCRs run for the confirmation of virulence genes detected in different pathotypes in this study.

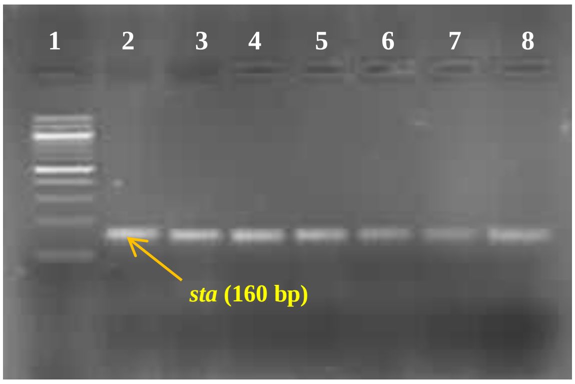

Figure S1: **Gel image of singleplex PCR products: Lane 1: 100bp molecular marker (ladder); Lane 2-8: *E. coli* isolates showing sta (160 bp) gene**

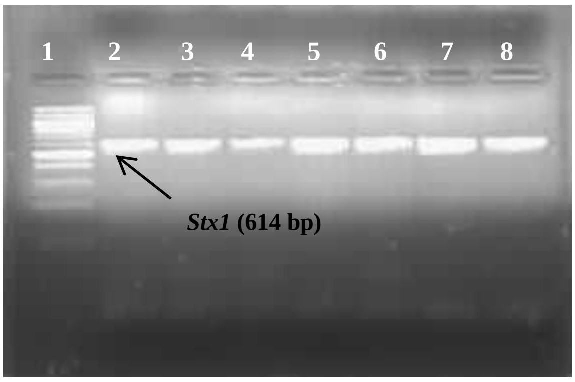

Figure S2: **Gel image of singleplex PCR products: Lane 1: 100bp molecular marker (ladder); Lane 2-8: *E. coli* isolates showing *Stx*1 (614 bp) gene**

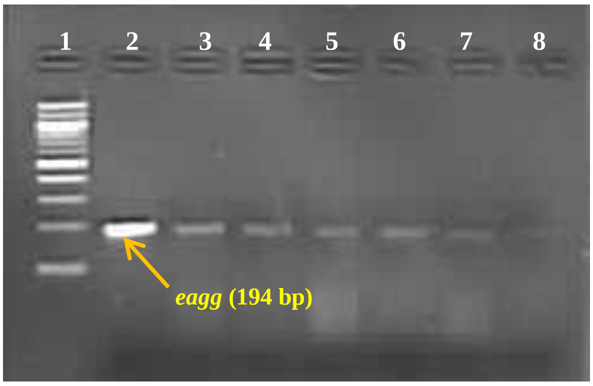

Figure S3: **Gel image of singleplex PCR products: Lane 1: 100bp molecular marker (ladder); Lane 2-8: *E. coli* isolates showing *eagg* (194 bp) gene (EAEC).**

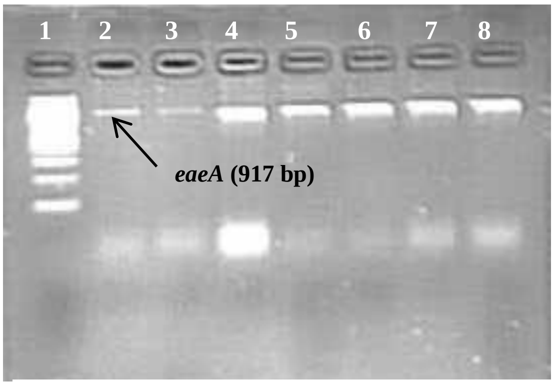

Figure S4: **Gel image of singleplex PCR products: Lane 1: 100bp molecular marker (ladder); Lane 2-8: *E. coli* isolates showing *eaeA* (917 bp) gene (aEPEC).**
